# CD36 Mediated Fatty Acid-Induced Podocyte Apoptosis via Oxidative Stress

**DOI:** 10.1371/journal.pone.0127507

**Published:** 2015-05-22

**Authors:** Wei Hua, Hui-zhe Huang, Lan-ting Tan, Jiang-min Wan, Hai-bo Gui, Liang Zhao, Xiong-zhong Ruan, Xue-mei Chen, Xiao-gang Du

**Affiliations:** 1 Department of Nephrology, The First Affiliated Hospital of Chongqing Medical University, Youyi Road 1, Chongqing, 400042, China; 2 Faculty of Basic Medical Sciences, Chongqing Medical University, Medical College Road 1, Chongqing, 400016, China; 3 Emergency Department, The First Affiliated Hospital of Chongqing Medical University, Youyi Road 1, Chongqing, 400042, China; 4 Centre for Nephrology, Royal Free and University College Medical School, University College London, Royal Free Campus, Rowland Hill Street, London, NW3 2PF, United Kingdom; 5 Centre for Lipid Research, Key Laboratory of Molecular Biology on Infectious Diseases, Ministry of Education, Chongqing Medical University, Youyi Road 1, Chongqing, 400042, China; 6 Laboratory of Lipid & Glucose Metabolism, The First Affiliated Hospital of Chongqing Medical University, Youyi Road 1, Chongqing, 400042, China; University of Houston, UNITED STATES

## Abstract

**Background:**

Hyperlipidemia-induced apoptosis mediated by fatty acid translocase CD36 is associated with increased uptake of ox-LDL or fatty acid in macrophages, hepatocytes and proximal tubular epithelial cells, leading to atherosclerosis, liver damage and fibrosis in obese patients, and diabetic nephropathy (DN), respectively. However, the specific role of CD36 in podocyte apoptosis in DN with hyperlipidemia remains poorly investigated.

**Methods:**

The expression of CD36 was measured in paraffin-embedded kidney tissue samples (Ctr = 18, DN = 20) by immunohistochemistry and immunofluorescence staining. We cultured conditionally immortalized mouse podocytes (MPC5) and treated cells with palmitic acid, and measured CD36 expression by real-time PCR, Western blot analysis and immunofluorescence; lipid uptake by Oil red O staining and BODIPY staining; apoptosis by flow cytometry assay, TUNEL assay and Western blot analysis; and ROS production by DCFH-DA fluorescence staining. All statistical analyses were performed using SPSS 21.0 statistical software.

**Results:**

CD36 expression was increased in kidney tissue from DN patients with hyperlipidemia. Palmitic acid upregulated CD36 expression and promoted its translocation from cytoplasm to plasma membrane in podocytes. Furthermore, palmitic acid increased lipid uptake, ROS production and apoptosis in podocytes, Sulfo-N-succinimidyloleate (SSO), the specific inhibitor of the fatty acid binding site on CD36, decreased palmitic acid-induced fatty acid accumulation, ROS production, and apoptosis in podocytes. Antioxidant 4-hydroxy-2,2,6,6- tetramethylpiperidine -1-oxyl (tempol) inhibited the overproduction of ROS and apoptosis in podocytes induced by palmitic acid.

**Conclusions:**

CD36 mediated fatty acid-induced podocyte apoptosis via oxidative stress might participate in the process of DN.

## Introduction

Diabetes mellitus (DM) has become a worldwide epidemic affecting nearly all developing and developed countries. Approximately 20 to 40% of patients with diabetes types 1 and 2, will develop diabetic nephropathy (DN), the main cause of chronic kidney disease (CKD), which ultimately progresses to end-stage renal disease (ESRD) associated with high costs substituting for kidney function [1]. Diabetic nephropathy is characterized by glomerular hypertrophy, widening of the glomerular basement membrane (GBM), mesangial expansion and podocytopenia, leading to nodular (Kimmelstiel-Wilson) glomerulosclerosis, which is called diabetic glomerulosclerosis [2].

Podocytes are highly branched, terminally differentiated visceral epithelial cells of the renal glomerulus that cover the urinary side of the GBM and play a crucial role in the regulation of glomerular function. Podocyte injury triggers proteinuria and glomerular sclerosis in human DN, and in animal models [3–5]. Reduction in podocyte number mediated by apoptosis has been shown to predict progressive decline in renal function and proteinuria in type 2 DM patients [6].

DM is often accompanied by lipid abnormalities. Experimental and clinical evidence suggests that lipid abnormalities in renal disease, including DN, contribute to the process of glomerulosclerosis with progressive decline in renal function [7–11]. Fatty acid translocase CD36 is expressed in several cell types, including macrophages, microvascular endothelial cells, platelets, adipocytes, and podocytes. Studies showed that hyperlipidemia-induced apoptosis mediated by CD36 was associated with increased uptake of ox-LDL or fatty acid in macrophages leading to atherosclerotic lesions[12], in hepatocytes leading to liver damage and fibrosis in obese patients[13], and in proximal tubular epithelial cells leading to progressive diabetic nephropathy[14]. However, the specific role of CD36 in podocyte apoptosis of DN with hyperlipidemia remains poorly investigated.

The aim of the present study was therefore, to investigate the expression of CD36 in kidney tissues from DN patients with hyperlipidemia, and the effect of fatty acid including the mechanism of action on CD36 expression and apoptosis of podocytes using an *in vitro* model.

## Materials and Methods

### Human kidney tissues

Paraffin-embedded kidney tissue samples (Ctr = 18, DN = 20) were collected from Pathology Department of the First Affiliated Hospital of Chongqing Medical University from January 2011 to December 2013, and the expression of CD36 was measured by immunohistochemistry and immunofluorescence. For control tissue samples, renal tissue was collected from 18 patients undergoing nephrectomy for renal trauma with no other evidence of renal disease. For DN tissue samples, renal tissue was collected from 20 DN patients with hyperlipidemia undergoing renal biopsies. The study was approved by the Ethics Committee of The First Affiliated Hospital of Chongqing Medical University and written informed consent was obtained from all the patients.

### Podocyte culture studies

Conditionally immortalized mouse podocyte cell line (MPC5) was kindly shared by Dr Ruan of the Centre for Nephrology, Royal Free and University College Medical School, London, United Kingdom. Cells were cultured and maintained in RPMI-1640 medium supplemented with 10% fetal bovine serum (FBS, Gibco), 1000 U/L penicillin, 1 mg/L streptomycin and 10U/ml interferon-γ on type I collagen at “permissive” temperature of 33°C and 5% carbon dioxide. Podocyte differentiation was induced by culturing them under “nonpermissive” temperature of 37°C without interferon-γ. All experiments were performed on podocyte cell line after differentiation for 14 days. Podocytes were treated with palmitic acid (Sigma-Aldrich) after pre-treatment with or without Sulfo-N-succinimidyloleate (SSO, Toronto Research Chemicals, North York, ON, Canada), a specific inhibitor of the fatty acid binding site on CD36, antioxidant4-hydroxy-2,2,6,6-tetramethylpiperidine-1-oxyl (tempol, Sigma-Aldrich), or vehicle (Dimethyl sulfoxide, DMSO). The CD36 expression, lipid uptake, reactive oxygen species (ROS) production and cell apoptosis were measured.

### Immunohistochemistry staining

Paraffin-embedded kidney tissue samples were sliced into 5-μm-thick sections, deparaffinized in xylene, and rehydrated in graded ethanol. Antigen recovery was performed in 10 mmol/L boiling sodium citrate buffer at pH 6.0 for 10 minutes at 92–98°C, and the specimens were incubated with 0.3% H_2_O_2_ for 15 minutes. Non-specific binding was blocked with normal goat serum for 10 minutes at room temperature. The sections were incubated with mouse monoclonal anti-CD36 antibody (diluted 1:100, Abcam, ab23680, UK) at 4°C overnight, washed three times with phosphate-buffered saline (PBS) and incubated with biotinylated secondary antibody (Zhongshan Golden Bridge Inc., China) for 10 minutes. After washing with PBS for three times, sections were incubated with DAB (3, 3’-diaminobenzidine) for 5 minutes, counterstained with hematoxylin for 5 seconds, visualized using Nikon Eclipse 80i microscope.

### Immunofluorescence staining of CD36

Tissue staining was accomplished by first slicing the paraffin-embedded kidney tissue samples into 5-μm-thick sections. After deparaffinization, rehydration, antigen recovery and blocking, the sections were incubated with mouse monoclonal anti-CD36 antibody (1:100) at 4°C overnight, and washed three times with PBS and incubated with secondary antibody (goat anti-mouse-IgG AlexaFluor 488, Invitrogen, A11001, USA) for 1.5 hours in light-shielded conditions. After three washes with PBS, sections were mounted with antifade reagents, coverslipped, and visualized microscopically.

Cultured cells were stained by plating the podocytes on glass coverslips. After treatment, the cells were washed three times with PBS and fixed with 4% paraformaldehyde for 10 minutes at room temperature. The cells were washed with PBS, blocked with 5% bovine serum albumin (BSA) for 1 hour at room temperature, and incubated with mouse anti-CD36 antibody (1:100) at 4°C overnight. The cells were washed three times with PBS and incubated with goat anti-mouse-IgG for 1.5 hours in light-shielded conditions. Nucleus was stained with 4, 6-diamidino-2-phenylindole (DAPI; Invitrogen, USA) for 2 minutes. After three washes with PBS, coverslips were mounted with antifade reagents. The cells were visualized microscopically.

### Real-time PCR

Total RNA was extracted by Bioteke Corporation Kit. First strand cDNAs synthesized from total RNA using PrimeScript RT reagent Kit (Takara, Japan) were used as templates. Specific primers were purchased from GeneCopoeia, USA. The levels of mRNA were quantified using All-in-One qPCR Mix Kit (GeneCopoeia, USA). Fold change for each group was determined using the delta-delta Ct method. Quantified mRNA levels were normalized to β-actin and presented relative to control group (podocytes were treated with 0 μmol/L palmitic acid for 0 hour).

### Western blot analysis

Cells were rinsed twice with PBS, sonicated for 15 seconds in 500 μl of RIPA lysis buffer (Beyotime, China), and centrifuged at 14,000g for 5 minutes. Protein concentration was then determined by bicinchoninic acid protein assay (Beyotime, Beijing, China). The sample loading buffer was added to the protein sample and heated at 100°C for 10 minutes. The proteins were separated by electrophoresis in 10% tris-glycine polyacrylamide gradient gels. The separated proteins were then transferred onto PVDF membrane (Millipore), blocked with 5% skimmed milk for 2 hours, and incubated with rabbit monoclonal anti-CD36 antibody (1:800, Abcam, ab133625, UK), or rabbit monoclonal cleaved-caspase3 antibody (1:1000, Cell Signaling Technology, 9664S, USA) overnight at 4°C. After washing and incubation with horseradish peroxidase (HRP)-labeled Goat anti-rabbit (1:8,000, MultiSciences, GAR007, China) or Goat anti-mouse IgG (1:8,000, MultiSciences, GAM007, China) at room temperature for 1.5 hours, the membranes were probed with chemiluminescence reagents using a commercially available kit (Pierce Biotechnology, USA). Protein expression was detected using a chemiluminescent staining reagent kit to visualize the signals, followed by exposure to x-ray films. Band intensities were quantified with Quantity One software, and calculated as the optical density×area of the band.

### Measurement of lipid uptake

Lipid uptake was measured using Oil Red O staining and BODIPY lipid probes. Oil Red O staining was carried out for 1 hour followed by 3 washes with distilled water. Lipid droplets were then visualized by microscope. To measure the uptake of fatty acid by BODIPY lipid probes, we incubated podocytes with 4, 4-difluoro-5-methyl-4-bora-3a,4a-diaza-s-indacene-3-dodecanoic acid (10 μg/ml, BODIPY500/510 C1, C12, Invitrogen, USA) at 37°C for 2 hours. Then the podocytes were washed with PBS to remove any extracellular fatty acid. Images were visualized under Olympus FluoView laser scanning confocal microscope (Olympus, Tokyo, Japan).

### Detection of ROS

Intracellular ROS level was measured using the 2′, 7′-dichlorofluorescein diacetate (DCFH-DA) fluorescent probe. Pretreated cells were reacted with DCFH-DA (10 μmol/L) at 37.0°C for 1 hour. Cell images were captured with fluorescence microscope (excitation wavelength was 488 nm and emission wavelength was 520 nm).

### Flow cytometry analysis

An apoptosis assay kit (Sungene Biotech, China) was used to determine apoptosis in confluent podocytes. Flow cytometry with BD FACSVantage SE cytometer was used to count cells that were annexin V-positive and propidium iodide-negative for early stages of apoptosis.

### Apoptosis detection by TUNEL assay

*In situ* detection of DNA fragmentation was performed using the ApoTag TUNEL assay following the manufacturer's protocol (Intergen, Purchase, New York, United States). Apoptotic nuclei were detected using PI staining (1 μg/ml) in cell cultures fixed with 4% paraformaldehyde, and the TUNEL-positive cells (Green) were analyzed via fluorescence microscopy. To quantify apoptosis, 400 nuclei from random microscopic fields were analyzed by an observer blinded to the treatment groups. The total number of apoptotic cells in each section was summed and expressed as the percentage of the total cell number. At least 10 individual sections were evaluated per slide. Each observer was blinded to other data concerning the cells, as well as to the results of the other observer.

### Statistical analysis

All statistical analyses were performed using SPSS 21.0 statistical software. All samples were analyzed in triplicate. Numeric data were shown as means ± SD. Statistical significance between groups was analyzed by Student's t-test and one-way ANOVA. *P* < 0.05 was considered statistically significant.

## Results

### Increased CD36 expression in kidney tissue of DN patients with hyperlipidemia

**Fig 1:**
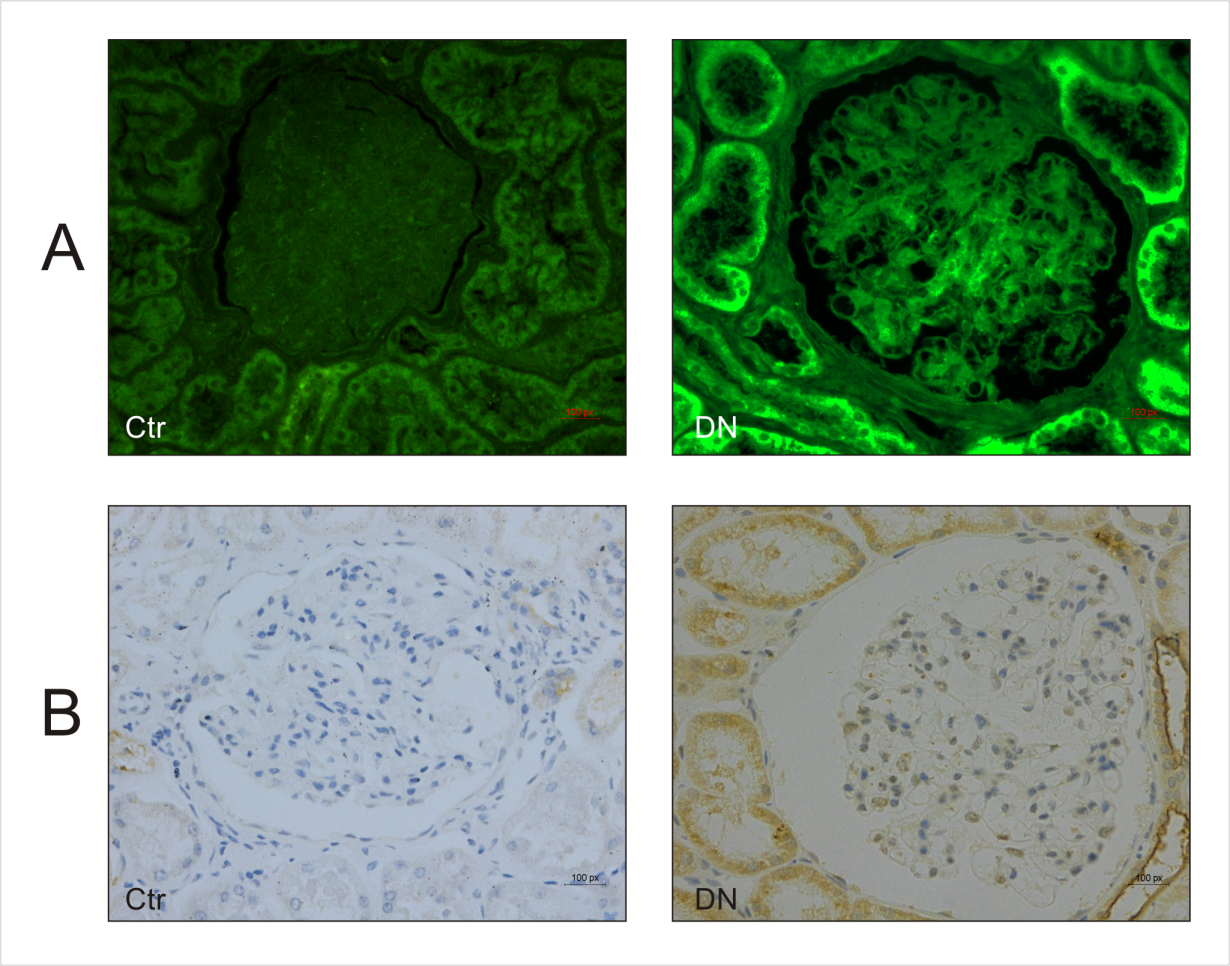
Renal tissue derived from DN patients with hyperlipidemia was characterized by increased expression of CD36. Immunofluorescence staining (A) and Immunohistochemical staining (B) of CD36 in renal tissue derived from control subjects or DN patients with hyperlipidemia (400×). Ctr: control. DN: Diabetes Nephropathy.

The CD36 expression was analyzed using immunofluorescence staining of kidney tissue samples collected from 20 DN patients with hyperlipidemia and 18 control patients without renal disease. As shown in Fig 1A, the expression of CD36 in both renal tubules and glomerulus of DN group increased significantly when compared with the control group. Similarly, immunohistochemical staining showed the expression of CD36 in DN group was markedly upregulated in comparison to the control group (Fig 1B).

### Palmitic acid upregulated CD36 expression of podocytes

**Fig 2:**
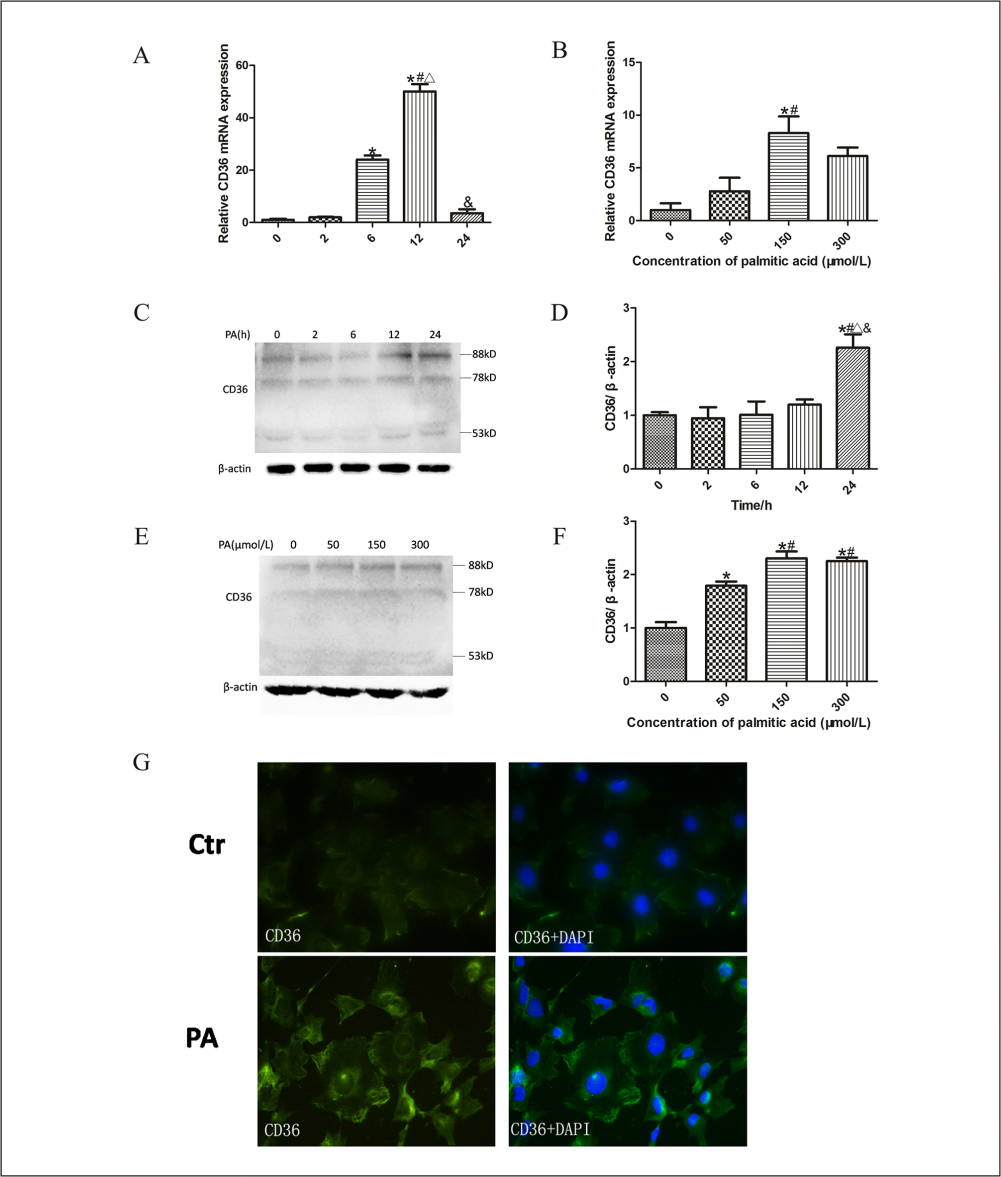
Palmitic acid increased CD36 expression in podocytes. Quantitative RT-PCR amplification of CD36 performed with RNA extracted from podocytes treated with 100μmol/L palmitic acid at different time points (A), or different concentration of palmitic acid for 12 hours (B). The fold change for each group was determined using the delta-delta Ct method. Quantified mRNA levels were normalized to β-actin and presented relative to control group (podocytes were treated with 0 μmol/L palmitic acid for 0 hour). Representative Western blots of CD36 protein expression in podocytes induced by 100μmol/L palmitic acid at different time points (C) and with different concentration for 24 hours (E). The relative band densities from Western blots of CD36 expression are shown in D and F, respectively. Data are presented as means ± SD from three independent experiments. * p < 0.05 vs.0h, # p < 0.05 vs. 2h, ∆ p < 0.05 vs. 6h, & p < 0.05 vs. 12h, (A, D). * p < 0.05 vs. 0 μmol/L, # p < 0.05 vs. 50 μmol/L (B, F). (G) Representative immunofluorescence staining of CD36. (400×). Ctr: control group, podocytes were treated with 1%BSA; PA: palmitic acid group, podocytes were treated with 150 μmol/L palmitic acid for 24 hours.

It has been reported that lipid disorders could stimulate the upregulation of CD36 in monocytes [15] and the translocation of CD36 from cytoplasm to cell membrane might play an important role in long-chain fatty acid (LCFA) uptake in adipocytes [16]. However, the effect of lipid disorders on CD36 expression in podocyte is still uncertain. We, therefore, treated podocytes with 100μmol/L palmitic acid for different durations and found that CD36 mRNA expression in podocytes increased and peaked at 12 hours (Fig 2A). The CD36 protein expression was significantly enhanced at 24 hours (Fig 2C and 2D), which demonstrated that high lipid levels upregulated CD36 expression in podocytes. Meanwhile, the dose-dependent increase of CD36 mRNA expression was shown in podocytes after exposure to palmitic acid for 12 hours (Fig 2B). CD36 protein expression was also markedly enhanced in podocytes treated with increasing dose of palmitic acid for 24 hours (Fig 2E and 2F). However, no significant difference of CD36 protein expression was found between podocytes treated with 150μmol/L and 300μmol/L palmitic acid for 24 hours, (Fig 2E and 2F). The results suggested that 150μmol/L was the appropriate dose of palmitic acid to induce CD36 expression of podocytes. Immunofluorescent staining further confirmed that palmitic acid increased CD36 expression in cell membrane and cytoplasm, especially in cell membrane (Fig 2G).

### Palmitic acid promoted lipid uptake of podocytes mediated by CD36

**Fig 3:**
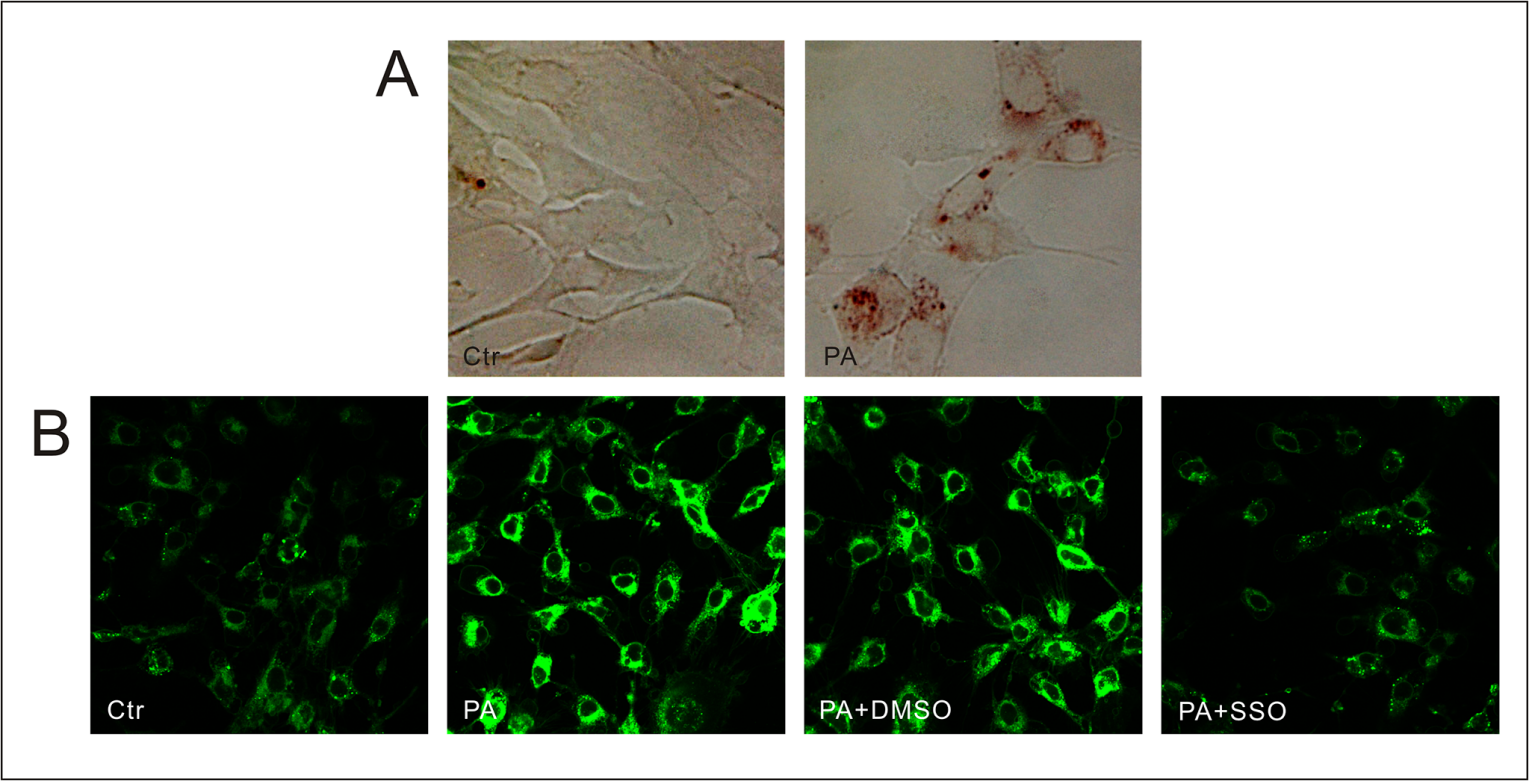
CD36 mediating lipid accumulation in cultured podocytes exposed to high fatty acid condition. (A) Lipid accumulation in control and podocytes after treatment with 150 μmol/L palmitic acid for 24 hours are stained by Oil Red (400×). (B) Representative immunofluorescence microscopy of fatty acid uptake in podocytes by BODIPY lipid probes (300×). Ctr: Control group, podocytes were treated with 1%BSA. PA: palmitic acid group, podocytes were treated with 150 μmol/L palmitic acid for 24 hours. PA+DMSO: podocytes were treated with 150 μmol/L palmitic acid for 24 hours after pretreatment with dimethyl sulfoxide (DMSO) for 4 hours. PA+SSO: podocytes were treated with 150 μmol/L palmitic acid for 24 hours after pretreatment with 50 μmol/L sulfo-N-succinimidyloleate (SSO) for 4 hours.

Using Oil Red O staining and BODIPY lipid probes, we found the increased lipid accumulation in podocytes incubated with palmitic acid (Fig 3). It has been found that fatty acid translocase CD36 was important for the uptake of lipids. SSO, an oleic acid derivative specifically inhibited lipid uptake of FAT/CD36 on the plasma membrane, resulting in an arrest of LCFA transport into cells [17, 18]. We pretreated podocytes with SSO followed by treatment with PA, and found that the increased lipid uptake in podocytes induced by palmitic acid was disrupted (Fig 3B), which confirmed that high lipid conditions promoted lipid uptake into podocytes mediated by CD36.

### Palmitic acid induced podocyte apoptosis mediated by CD36

**Fig 4:**
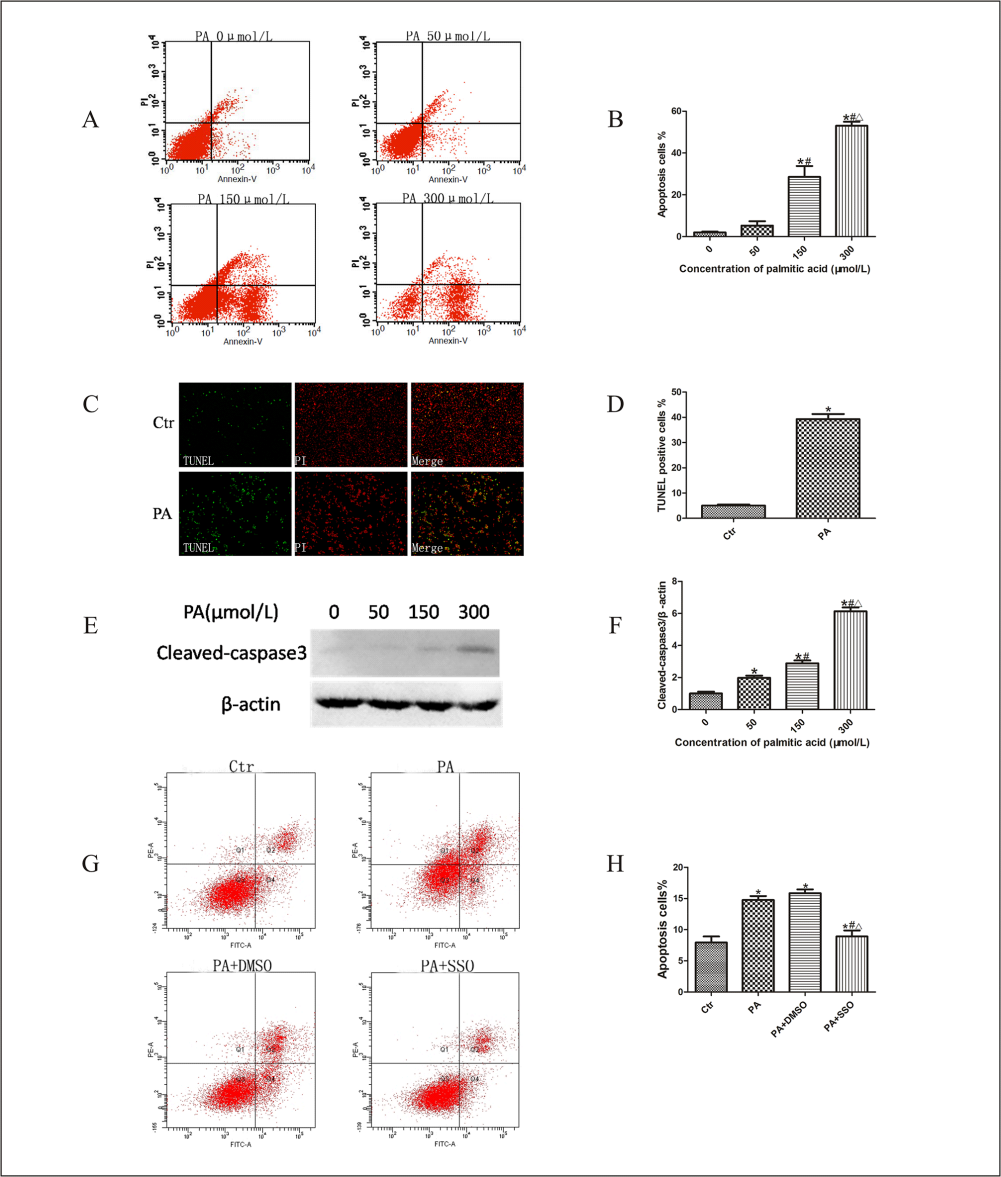
CD36 mediated palmitic acid-induced podocyte apoptosis. **(A, B) Podocytes were treated with different concentrations of palmitic acid for 24 hours, apoptosis was detected using flow cytometry analysis.** (A) Representative cytograms. (B) Percentage of apoptotic cells. * p < 0.05 vs. 0 μmol/L, # p < 0.05 vs. 50 μmol/L. ∆ p < 0.05 vs. 150μmol/L. (C, D) Podocytes were treated with 150 μmol/l palmitic acid for 24 hours, apoptosis was measured using TUNEL and PI staining assay. (C) Representative immunofluorescence microscopy. (D) Percentage of apoptotic cells. * p < 0.05 vs. control group. (E) Representative Western blots of cleaved-caspase3 expression of podocytes treated with different concentration of palmitic acid for 24 hours. (F) Relative band densities from Western blots of cleaved-caspase3 expression of podocytes after treatment with different concentration of palmitic acid. * p < 0.05 vs. 0μmol/L, # p < 0.05 vs. 50 μmol/L, ∆ p < 0.05 vs. 150 μmol/L. (G, H) Podocytes were treated with 150μmol/L palmitic acid for 24 hours with or without pretreatment of SSO or DMSO, apoptosis was measured using flow cytometry analysis. (G) Representative cytograms. (H) Percentage of apoptotic cells detected by flow cytometry was shown; * p < 0.05 vs. control group, # p < 0.05 vs. PA group, ∆ p < 0.05, vs. PA+DMSO group. Data are expressed as mean ± SD. Ctr: Control group, podocytes were treated with 1%BSA. PA: palmitic acid group, podocytes were treated with 150 μmol/L palmitic acid for 24 hours. PA+DMSO: podocytes were treated with 150 μmol/L palmitic acid for 24 hours after pretreatment with dimethyl sulfoxide (DMSO) for 4 hours. PA+SSO: podocytes were treated with 150μmol/L palmitic acid for 24 hours after pretreatment with 50 μmol/L of sulfo-N-succinimidyloleate (SSO) for 4 hours.

To study the effect of palmitate on podocyte apoptosis *in vitro*, we treated podocytes with palmitic acid. Flow cytometry revealed that apoptosis rates significantly increased when treated with palmitic acid at concentrations of 150 and 300 μM (Fig 4A and 4B). TUNEL and PI staining was employed to further validate the apoptotic effect of palmitic acid on podocytes. Massively increased apoptotic cells were observed in the palmitic acid group (39.3%) in contrast to control group (5.1%) (Fig 4C and 4D) clearly indicating that palmitic acid induced podocyte apoptosis.

The activation of caspase-3, an important kinase protein that plays a key role in the regulation of cell apoptosis, is believed to stimulate mitochondrial cell death signals [19]. In this study, we investigated the effect of palmitic acid on the activation of caspase-3 by Western blot using specific cleaved-caspase3 antibody. In Fig 4E and 4F, the Western blot results showed dose-dependent upregulated expression of cleaved-caspase-3 in palmitic acid-treated podocytes. These results demonstrated that treatment with palmitic acid significantly induced dose-dependent apoptosis in podocytes.

To further investigate whether CD36 participated in lipid-induced podocyte apoptosis, we treated podocytes with palmitic acid after pretreatment with SSO, and found that apoptosis was inhibited (Fig 4G and 4H), compared with those in PA group (podocytes treated with palmitic acid) or PA+DMSO group (podocytes were treated with150μmol/L palmitic acid for 24 hours after pretreatment with DMSO for 4 hours). Taken together, these data provided evidence that palmitic acid induced podocyte apoptosis was mediated by CD36.

### Oxidative stress in fatty acid-induced podocyte apoptosis mediated by CD36

**Fig 5:**
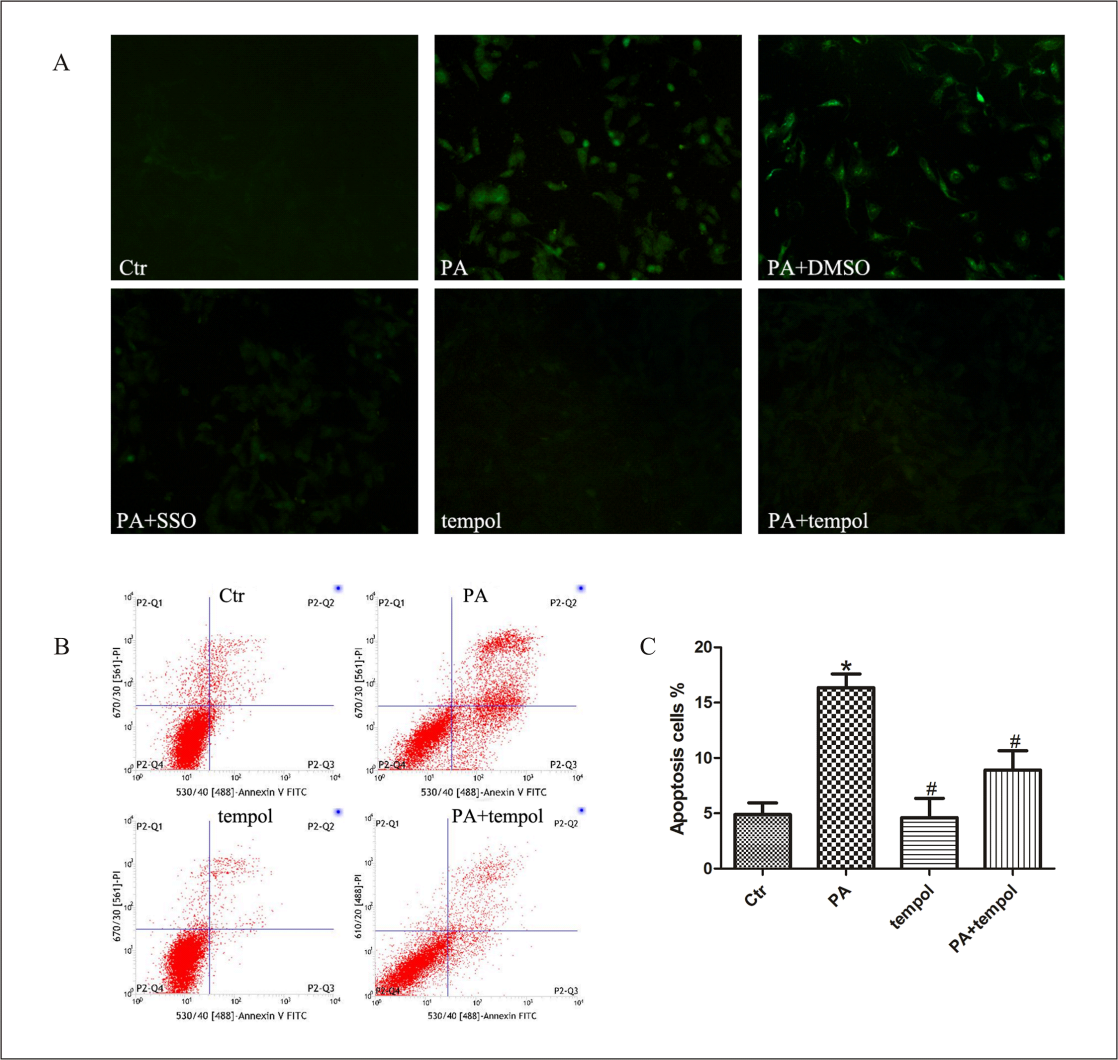
Oxidative stress in fatty acid-induced podocyte apoptosis mediated by CD36. (A) Representative immunofluorescence microscopy of ROS in podocytes (200×). Podocytes were treated with 150 μmol/L palmitic acid for 24 hours with or without pretreatment of SSO or tempol, then intracellular ROS level was measured using 2′,7′- dichlorofluorescein diacetate (DCFH-DA) fluorescent probe (green). (B, C) Podocytes were treated with 150 μmol/L palmitic acid for 24 hours with or without pretreatment using tempol, and apoptosis was measured using flow cytometry analysis. (B) Representative cytograms. (C) Percentage of apoptotic cells. * p < 0.05 vs. control group, # p < 0.05 vs. PA group. Data were expressed as mean ± SD. Ctr: Control group, podocytes were treated with 1% BSA. PA: palmitic acid group, podocytes were treated with 150 μmol/L palmitic acid for 24 hours. PA+DMSO: podocytes were treated with 150 μmol/L palmitic acid for 24 hours after pretreatment with dimethyl sulfoxide (DMSO) for 4 hours. PA+SSO: podocytes were treated with 150 μmol/L of palmitic acid for 24 hours after pretreatment with 50 μmol/L sulfo-N-succinimidyloleate (SSO) for 4 hours. tempol: podocytes were treated with 1% BSA for 24 hours after pretreatment with 0.5 mmol/L of tempol for 2 hours. PA+ tempol: podocytes were treated with 150 μmol/L palmitic acid for 24 hours after pretreatment with 0.5 mmol/L of tempol for 2 hours.

Using the reactive oxygen species (ROS)-sensitive fluorescent probe 2′, 7′-dichlorofluorescein diacetate (DCFH-DA) to monitor cellular oxidative stress, we found that ROS production was considerably increased in podocytes treated with palmitic acid when compared with control group, and inhibition of CD36 function with SSO decreased the production of ROS induced by pamitate (Fig 5A). Furthermore, we found that antioxidant tempol significantly attenuated enhanced ROS production (Fig 5A) and apoptosis (Fig 5B and 5C) in podocytes treated with palmitic acid, confirming that oxidative stress in podocytes induced by fatty acid was mediated by CD36.

## Discussion

DN, one of the microvascular complications of diabetes, is the most common cause of CKD worldwide, with a prevalence of 40% in patients with ESRD[20]. Hyperglycemia-associated increased formation of intracellular advanced glycation end-products (AGEs) and activation of protein kinase C isoforms [21, 22], systemic and glomerular hypertension, and activation of renin-angiotensin system (RAS) are major factors in the pathogenesis of DN. Current therapies for DN focus on controlling blood sugar and blood pressure, and inhibiting the renin-angiotensin system (RAS) to reduce proteinuria, in an effort to delay the progression of DN [23, 24]. However, with increasing incidence of DN and deteriorating kidney function resulting in ESRD, it is imperative to understand the pathogenesis of DN comprehensively and identify effective therapeutic interventions and strategies.

It has been found that DM is often complicated with dyslipidaemia associated with elevated triglycerides (TG) [25]. Hyperlipidemia is associated with DN progression, and lipid-lowering agents might protect renal function [7]. CD36 is a transmembrane protein of the class B scavenger receptor family, a broadly expressed membrane glycoprotein mediating the uptake of LCFA in adipocytes and fibroblasts, in arteriosclerosis, fatty liver, obesity and inflammatory responses, and so on[12, 13, 26–28]. In present study, using immunofluorescence and immunohistochemical staining we found an elevated expression of CD36 in the kidney of DN patients with hyperlipidemia, suggesting that CD36-mediated renal uptake of lipids might be associated with DN.

The CD36 cDNA predicts a polypeptide of 53-kDa composed of 471 amino-acid residues. After variable post-translational N-linked glycosylations, which are necessary for trafficking to the plasma membrane, CD36 protein presents different molecular masses (78, 88 or 94 KDa) corresponding to different glycoforms [29]. LCFA uptake in adipocytes requires plasma membrane rafts, and FAT/CD36 which recycles from intracellular nonlipid raft domains to lipid raft regions of the plasma membrane might control LCFA uptake [16]. In the present study, treating podocytes with palmitic acid increased the CD36 expression in podocytes at both transcriptional and protein levels (53KDa, 78KDa and 88KDa), in the cell membrane and cytoplasm. Further research showed palmitate induced lipid accumulation in podocytes, and SSO, which specifically binds to and cross-links FAT/CD36 on the plasma membrane without permeating the plasma membrane, disrupted the lipid uptake. These results confirmed that palmitate not only upregulated CD36 expression but also promoted CD36 translocation from cytoplasm to plasma membrane in podocytes, leading to lipid accumulation in podocytes.

DN is characterized by early-stage microalbuminuria and an increasing magnitude of proteinuria. Massive proteinuria is an adverse prognostic factor in chronic renal failure (CRF) irrespective of etiology, and interventions designed to minimize excessive glomerular protein filtration may halt or slow the loss of function at early and late stages of CRF [30, 31]. Podocyte apoptosis has been suggested as a potent mechanism of proteinuria in diabetic nephropathy [32]. Additional studies are required to investigate the detailed mechanisms of podocyte apoptosis. We treated podocytes with PA, and found increased apoptosis, which was inhibited by SSO, a specific inhibitor of lipid uptake mediated by FAT/CD36 on the plasma membrane, suggesting that CD36 might play a key role in podocyte apoptosis induced by FFA.

Fatty acids and triglyceride-rich emulsions stimulate ROS production in leukocytes [33]. Oxidative stress plays a pathological role in the development of various diseases including DM and atherosclerosis [34], and an increase in podocyte ROS levels was a potential mediator of podocyte apoptosis in DM [6]. Here, we treated podocytes with PA, and found ROS production was considerably increased. Inhibition of ROS overproduction by tempol largely abrogated podocyte apoptosis stimulated by palmitic acid, suggesting that oxidative stress played an important role in fatty acid-induced podocyte apoptosis. We further confirmed that pretreatment of SSO decreased ROS production induced by PA in podocytes. Thus, oxidative stress might participate in CD36-mediated palmitic acid-induced podocyte apoptosis. The inhibition of ROS by modulating CD36 expression and translocation might be part of a protective mechanism against saturated FFAs that drives podocyte apoptosis. However, the exact mechanism of action and regulation of CD36 involved in fatty acid-inducing podocyte apoptosis is not well understood and is the subject of ongoing investigations.

In summary, we have shown that CD36 expression was increased in kidney tissue of DN patients with hyperlipidemia. Palmitic acid upregulated CD36 expression and promoted CD36 translocation from cytoplasm to plasma membrane in podocytes. Increased lipid uptake, ROS production and apoptosis in podocytes induced by palmitic acid were mediated by CD36. All of these suggested CD36 mediated fatty acid-induced podocyte apoptosis via oxidative stress might participate in the process of DN. Further studies are required to determine the exact mechanism of action and regulation of CD36 involved in fatty acid-induced podocyte apoptosis.
